# Comparative Genomics Analysis and Pathogenicity Assessment of *Colletotrichum siamense* Strain JDA-12 on Multiple Strawberry Cultivars

**DOI:** 10.3390/plants15152366

**Published:** 2026-07-31

**Authors:** Dong Liang, Xiao-Qi Yang, Xiao-Lu Wu, Chuan-Qing Zhang

**Affiliations:** 1 College of Advanced Agricultural Sciences, Zhejiang Agriculture and Forest University, Hangzhou 311300, China; liangdong@zafu.edu.cn (D.L.); yangxiaoqi1232020@163.com (X.-Q.Y.); 2 Hangzhou Chenxin Ecological Agriculture Development Co., Ltd., Hangzhou 311500, China; m13656682951@163.com

**Keywords:** strawberry crown rot, *Colletotrichum siamense* strain JDA-12, comparative genomics, pathogenicity profile, environment factors

## Abstract

Strawberry crown rot (SCR) disease poses a significant threat to strawberry production in Zhejiang Province, China. In our previous study, we identified *Colletotrichum siamense* strain JDA-12 as the dominant causal agent of SCR disease in samples collected from Zhejiang. However, the factors associated with this phenomenon remain unclear. In this study, a comparative analysis of JDA-12 and other documented *Colletotrichum* species revealed that the gene families AbiEii and CBM24-GH71, together with two annotated RiPP-like secondary metabolite biosynthetic clusters, are specifically expanded or uniquely present in JDA-12. These lineage-specific genomic features may represent genomic signatures associated with ecological adaptation and host-associated interactions of JDA-12. Additionally, we assessed the pathogenicity of JDA-12 on major strawberry cultivars grown in Zhejiang. The findings demonstrated that ‘Benihoppe’ exhibited high susceptibility, while ‘Fenyu No.1′ showed comparatively strong resistance. Furthermore, we evaluated the impact of environmental factors on pathogenicity and found that short-day conditions (10L:14D), high temperature (~30 °C), and high humidity (≥90%) constitute the optimal environment for JDA-12 infection in strawberries, at least under conditions representative of Zhejiang. This study provides genomic insights into the ecological adaptation and pathogenic potential of JDA-12, while revealing environmental conditions favorable for SCR development, facilitating the development of effective disease management strategies.

## 1. Introduction

The cultivated strawberry (*Fragaria × ananassa*), a high-value crop belonging to the Rosaceae family, originates from South America and is globally esteemed for its nutrient-rich fruits, which are abundant in phytonutrients such as phenolics, fiber, and vitamins [1]. Its production has expanded across all continents, establishing itself as a vital sector in horticulture and agricultural economies [2]. Over the past three decades, both global yield and market value have consistently increased [3], reaching $15.3 billion in 2023 and projected to exceed $18.6 billion by 2028, with China leading in production since 1994. Strawberry production seasons vary across regions, depending on climatic conditions and cultivation systems. In the United States, production peaks during spring and early summer, while in China and Japan, economically valuable short-day cultivars are primarily cultivated under protected conditions during winter and spring [4]. However, disease pressures increasingly threaten strawberry yield and fruit quality, presenting significant challenges to sustainable production.

The southeastern region of China, encompassing the provinces of Zhejiang, Jiangsu, Shanghai, and Anhui, is recognized as one of the major strawberry-producing areas in the country [4]. Unfortunately, the hot and humid subtropical climate of this region creates favorable conditions for the development of strawberry crown rot (SCR), which poses a significant threat to strawberry production. SCR disease is characterized by symptoms such as lesions on stolons and petioles, as well as irregular leaf spots, particularly in plant nurseries [5]. In recent years, the severity of SCR disease has escalated in China, resulting in crown infections that lead to seedling mortality rates of up to 50% in nurseries and field yield losses exceeding 40% [6,7,8].

In recent decades, the etiology of SCR disease has been primarily attributed to species within the *Colletotrichum gloeosporioides* species complex (CGSC) and the *Colletotrichum acutatum* species complex (CASC) [9,10]. While CGSC was previously regarded as the primary agent of SCR disease [11], an increasing body of research indicates that *Colletotrichum siamense* is the true causal agent of strawberry diseases, supported by accumulating evidence from phylogenetic analyses and pathogenicity assays [12,13]. Recent studies from Hubei, China, and North Carolina, USA, have consistently identified *C. siamense* as the causative agent of severe SCR disease, with its potential resistance to certain fungicides raising serious concerns for effective disease management. Furthermore, surveys in China have confirmed the predominance of *C. siamense* (45.9%) and *C. fructicola* (38.4%), particularly in Zhejiang and Hubei provinces [14,15]. Similar trends have also been reported in Brazil and the United States. However, the specific mechanisms underlying the host adaptation of *C. siamense* to strawberries remain poorly understood.

Fungi with distinct genomic architectures exhibit extensive physiological diversity, employing species-specific strategies and specialized molecular mechanisms for host and environmental adaptation [16]. Information encoded within the fungal genome is ultimately manifested at the cellular level as functional traits, thereby influencing their life cycles and infection strategies [17]. Given the genomes of numerous *Colletotrichum* species, including various *C. siamense* strains isolated from diverse host plants cultivated in different countries, the objective of the present study is to investigate genomic characteristics potentially associated with ecological adaptation and host colonization of *C. siamense* in strawberry-associated environments by comparing it to closely related species.

In the present study, we isolated and identified pathogens from the crown tissues of diseased strawberry plants collected across Zhejiang Province, China, and identified *C. siamense* strain JDA-12 as the predominant species. Genome sequencing revealed that JDA-12 possesses the largest genome size and the highest number of gene models among previously characterized *C. siamense* strains. Comparative genomic analyses highlighted expansions in specific gene families, including AbiEii- and CBM24-GH71-containing proteins, as well as the enrichment of RiPP-like secondary metabolite clusters. These lineage-specific genomic features may contribute to ecological adaptation and host-associated interactions of JDA-12, although their biological functions require further experimental validation. Furthermore, we constructed a GFP-labeled transformant of JDA-12 to assess its pathogenicity across multiple strawberry cultivars. The strain exhibited the highest virulence on ‘Benihoppe’ and the lowest on ‘Fenyu No.1′. Environmental assays further demonstrated that short photoperiods, elevated temperatures, and high humidity significantly enhanced infection. These findings provide new insights into the genomic basis and environmental responsiveness of *C. siamense* JDA-12 pathogenicity in strawberries.

## 2. Results

### 2.1. Pathological Features of Crown Rot in Strawberry Caused by Colletotrichum

**Figure 1:**
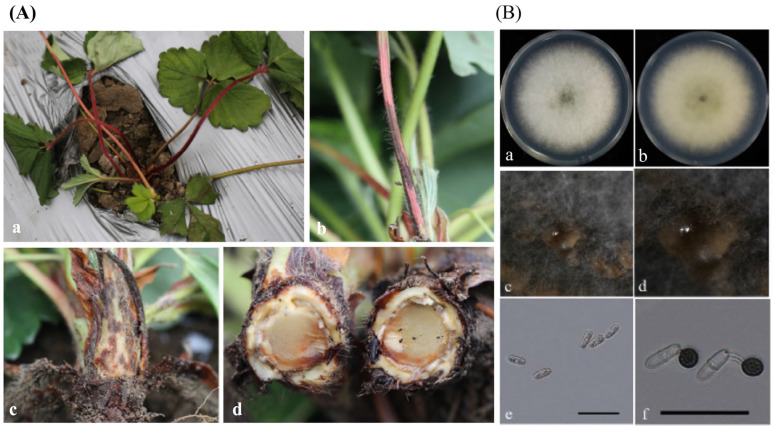
Strawberry crown rot (SCR) disease caused by *C. siamense* strain JDA-12 (**A**). panel (**A**(**a**)), the symptoms of the entire diseased strawberry plant; panel (**A**(**b**)), the symptoms of the stolons; panel (**A**(**c**)), the symptoms of the crown; panel (**A**(**d**)), the symptoms of the internal part of the crown. (**B**). panel (**B**(**a**,**b**)), colony morphology diagrams on PDA medium; panel (**B**(**c**,**d**)), conidia; panel (**B**(**e**)), Spore morphology; panel (**B**(**f**)), appressoria morphology (20 = μm).

Typical crown rot symptoms were observed in strawberry plants in the field (Figure 1A(a)). Dark brown to black necrotic lesions were visible on petioles and crown tissues (Figure 1A(b,c)). Longitudinal and transverse sections of infected crowns showed internal vascular discoloration and tissue necrosis (Figure 1A(c,d)). The fungal isolates produced white to pale gray colonies on PDA after incubation (Figure 1B(a,b)). Orange conidial masses developed on the colony surface under humid conditions (Figure 1B(c,d)). Microscopic observation showed that conidia were hyaline, cylindrical, and aseptate, consistent with morphological characteristics of *Colletotrichum* spp. (Figure 1B(e,f)). In our previous study [14], we isolated 150 *Colletotrichum* spp. from crown lesions, among which the *C. siamense* strain JDA-12 exhibited the highest pathogenicity and the most stable growth phenotype. To investigate the genetic basis of pathogenicity, we performed whole-genome sequencing of JDA-12, followed by a detailed analysis of virulence-associated gene families, as described below.

### 2.2. Phylogeny and Genomic Characteristics of C. siamense JDA-12 and Related Species

**Figure 2:**
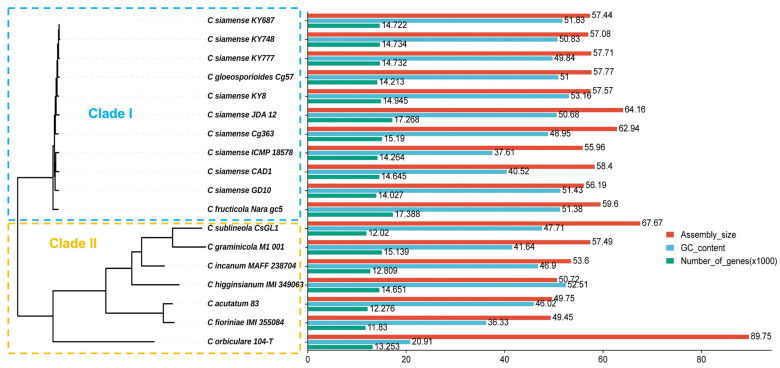
Genomic features of *Colletotrichum* species analyzed in this study. A maximum likelihood phylogenetic tree was constructed from all single-copy orthologous genes with 1000 bootstrap replicates. Red bars indicate genome size (Mb), blue bars show GC content (%), and green bars represent the number of predicted gene models in each species.

The whole genome of JDA-12 was sequenced and assembled using a hybrid strategy integrating Oxford Nanopore Technologies (ONT) long reads and Illumina paired-end short reads. Genome assemblies and annotations of 17 additional *Colletotrichum* species were retrieved from the NCBI database (see Table 1). The quality of the assemblies varied significantly, with scaffold counts ranging from 13 to 2896 and N_50_ values between 77,820 bp and 6.44 Mb. The genome sizes ranged from 49.13 Mb to 89.75 Mb, with JDA-12 comprising 64.16 Mb distributed across 285 scaffolds, a maximum scaffold length of 7.93 Mb, and an N50 value exceeding 4.50 Mb (Figure 2). Genome completeness was evaluated using BUSCO v5.12, based on the Ascomycota lineage dataset, which revealed high completeness (92.8–99.3%) across the species (Figure S1). Notably, JDA-12 achieved a completeness of 98.3%, confirming the robustness of the assembly. The predicted gene models varied from 11,830 to 17,388 (mean = 14,399), and JDA-12 encoded 17,268 genes.

Phylogenetic analysis based on single-copy orthologs resolved the species into two major clades (Figure 2). Clade I included multiple strains of *C. siamense*, along with *C. gloeosporioides* and *C. fructicola*, which are members of the *C. gloeosporioides* species complex (CGSC) [18]. While *C. gloeosporioides* and *C. siamense* clustered closely, *C. fructicola* formed a distinct branch, indicating divergent adaptive evolution. JDA-12 was most closely related to *C. siamense* KY8 (from apple, USA) and Cg363 (from strawberry, Japan). Clade II comprised more distantly related taxa, such as *C. acutatum*, *C. higginsianum*, and *C. graminicola*, reflecting divergent evolutionary or ecological trajectories. GC content analysis further elucidated clade-specific distinctions: Clade I species exhibited GC contents ranging from 37.61% to 53.16% (mean = 48.84%), which were substantially higher than those in Clade II (20.91–52.51%, mean = 41.72%) (Table 1; Figure 2).

### 2.3. Ortholog Identification in Colletotrichum Species Reveals Rapidly Expanded Orthologs in C. siamense JDA-12

**Figure 3:**
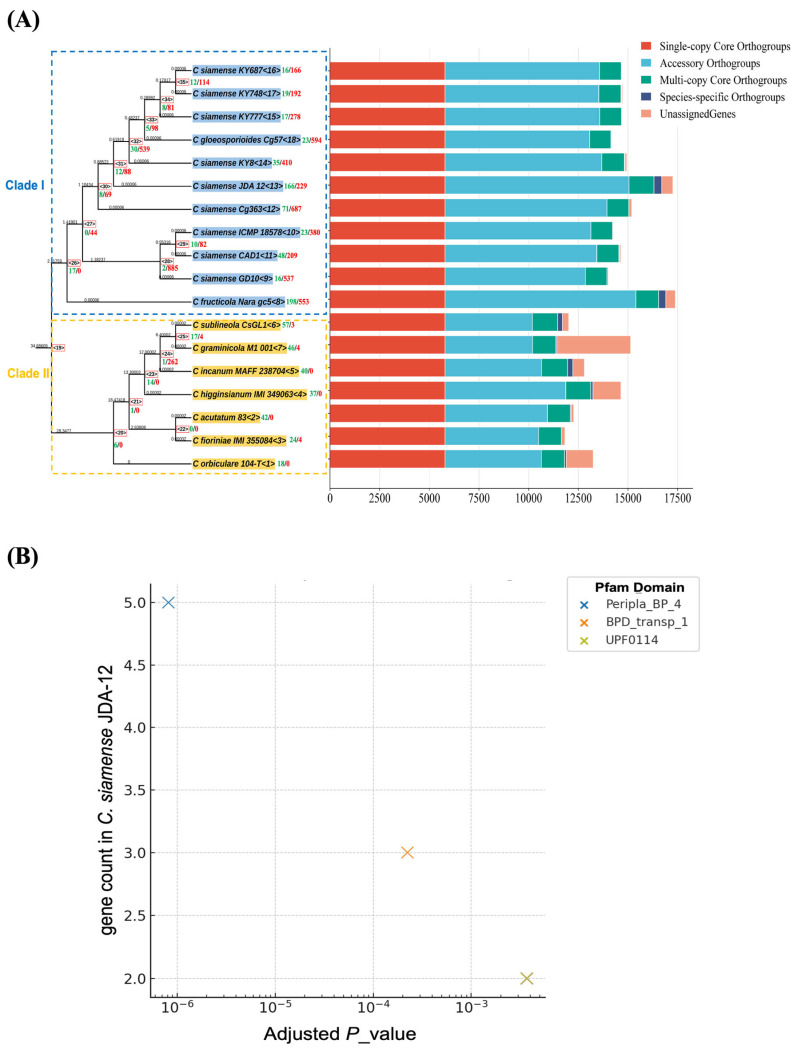
Ortholog analysis and identification of rapidly evolving orthologs. (**A**) Phylogenic analysis and rapidly evolving orthologs of *Colletotrichum* species in present study. Green numbers represent counts of expanded orthologs, and red numbers represent contracted orthologs. (**B**) Domain enrichment analysis of orphan proteins identified in *C. siamense* JDA-12. The colored ‘x’ markers represent three enriched Pfam domains in orphan proteins: blue ‘x’ for Periplasmic binding protein domain 4 (Peripla_BP_4, PF13407), orange ‘x’ for Binding-protein-dependent transport system inner membrane component domain 1 (BPD_transp_1, PF00528), and yellow ‘x’ for Uncharacterized protein family UPF0114 (UPF0114, cl01078).

An analysis of orthologous relationships among 258,106 proteins from 18 *Colletotrichum* species were conducted, resulting in 28,040 orthogroups. Out of these, 18,777 orthogroups were successfully assigned, while 9263 proteins remained unassigned as singletons (Table S1). A robust phylogenetic relationship was constructed based on 5827 single-copy orthogroups (Figure 3A; Table 2). Additionally, 995 multi-copy core orthologs (present in all species but variable in copy number) were identified, with gene counts ranging from 1034 in *C. gloeosporioides* Cg57 to 1311 in *C. incanum* MAFF 238704. This suggests lineage-specific gene expansion events. A total of 11,579 accessory orthologs, shared by at least two but not all species, displayed significant variation, ranging from 4399 genes in *C. sublineola* CsGL1 to 9611 in *C. fructicola* Nara_gc5, indicating their potential roles in niche-specific adaptation. Moreover, 415 species-specific orthogroups were identified. JDA-12 possessed the highest number (n = 392), whereas no expansion was detected in *C. fioriniae* IMI 355084. The number of unassigned genes varied widely (from 24 to 3716), potentially reflecting species-specific orphan gene repertoires. Pfam domain annotation revealed that 712 of the 9263 unassigned genes (7.7%) encoded recognizable functional domains. Notably, domain enrichment analysis of orphan genes in JDA-12 identified the Periplasmic binding protein domain 4 (Peripla_BP_4; PF13407) as the only significantly enriched domain (adjusted *p*-value = 0.00046, Fisher’s exact test), with five domain-containing proteins exclusively present in JDA-12 (Figure 3B; Table S2).

**Figure 4:**
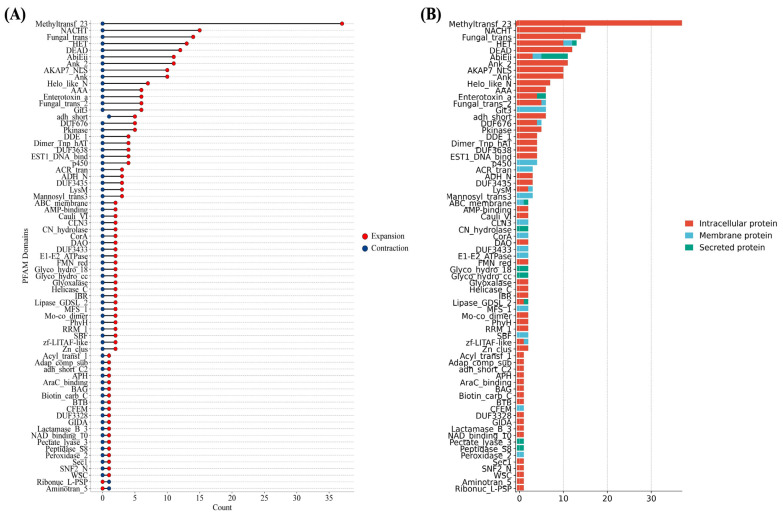
Functional classification of proteins assigned to rapidly evolved orthologs in the JDA-12 genome. (**A**) Pfam domain annotation of proteins from expanded (red dots) and contracted (blue dots) ortholog groups. The x-axis indicates the number of proteins containing each corresponding Pfam domain. (**B**) In silico subcellular analysis of proteins from rapidly evolved orthologs. The x-axis represents the count of proteins predicted in each localization category.

To investigate gene family evolution, we employed CAFE v5 to detect rapidly evolving orthologs. Using a λ parameter of 0.0001 and a Viterbi *p*-value threshold of <0.01, 17 rapidly expanded orthogroups were identified at the internal node <26>, which represents the most recent common ancestor (MRCA) of clade I species. Similarly, six expanded orthogroups were observed at the MRCA of clade II. Notably, extant species exhibited significantly more rapidly evolving orthogroups than their ancestral nodes (Mann–Whitney U test, *p*-values = 0.046), suggesting post-divergence expansion events. CAFE analysis inferred that the ancestral node (<31>) underwent relatively few gene family contractions but multiple expansion events. This expansion-dominated evolutionary pattern may partly explain the larger genome gene content observed in C. siamense JDA-12 relative to closely related species. Functional annotation of significantly expanded orthogroups in JDA-12 revealed the top ten enriched Pfam domains (Figure 4): Methyltransf_23 (PF13489), NACHT (PF05729), Fungal_trans (PF04082), HET (PF06985), DEAD (PF00270), AbiEii (PF08843), Ank_2 (PF12796), AKAP7_NLS (PF10469), Ank (PF00023), and Helo_like_N (PF17111). Subcellular localization analysis was performed using SignalP 6.0 for N-terminal secretion signal prediction and TMHMM 2.0 for transmembrane helix prediction. Among the expanded AbiEii-containing proteins, 11 proteins were predicted to contain secretion signals without transmembrane domains, suggesting their potential secretion.

### 2.4. Secretome Profiling and Analysis of Colletotrichum Species

**Figure 5:**
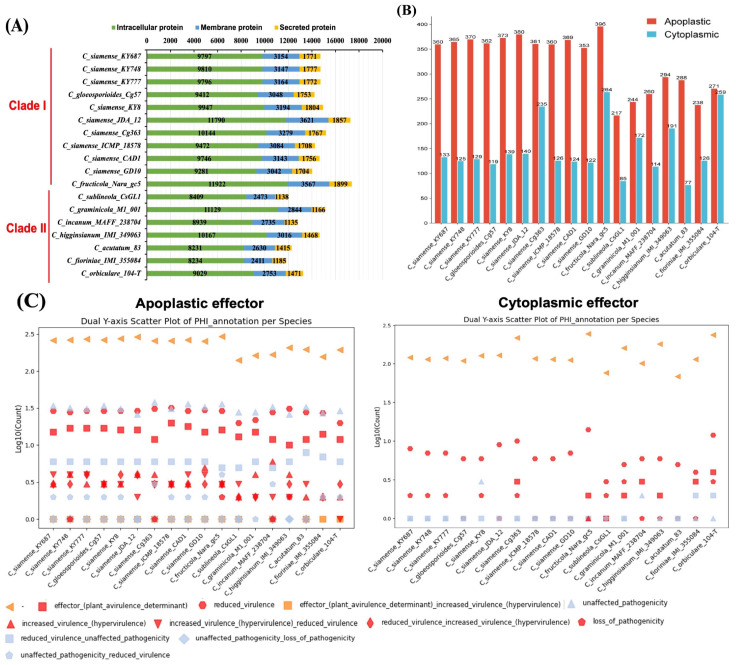
Comparative analysis of the secretome across *Colletotrichum* genomes presented in this study. (**A**) Numbers of predicted intracellular (green), membrane-associated (blue), and secreted (yellow) proteins in each species. (**B**) Predicted apoplastic and cytoplasmic effector candidates per species. Red and blue bars denote apoplastic and cytoplasmic effectors, respectively. (**C**) Distribution of PHI-base phenotype annotations for apoplastic (**left**) and cytoplasmic (**right**) effectors. Scatter plots display log-transformed effector counts for different PHI virulence phenotypes represented by specific shapes and colors: red right-pointing triangle, effector (plant avirulence determinant); red circle, reduced virulence; orange square, effector (plant avirulence determinant)_increased virulence (hypervirulence); light blue triangle, unaffected pathogenicity; red upward triangle, increased virulence (hypervirulence); red downward triangle, increased virulence (hypervirulence)_reduced virulence; red diamond, reduced virulence_increased virulence (hypervirulence); red hexagon, loss of pathogenicity; light blue square, reduced virulence_unaffected pathogenicity; light blue diamond, unaffected pathogenicity_loss of pathogenicity; orange downward triangle, reduced virulence_unaffected pathogenicity_loss of pathogenicity.it.

**Figure 6:**
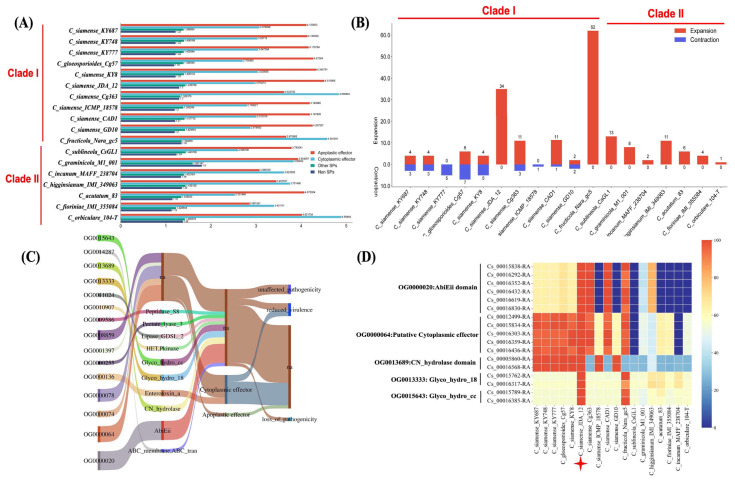
Integrated analysis of secreted proteins assigned to orthologs expanded in JDA-12. (**A**) Cysteine content (%) of predicted apoplastic effectors (red), cytoplasmic effectors (blue), other secreted proteins (green), and non-secreted proteins (dark blue). (**B**) Numbers of secreted proteins belonging to orthologs expanded (red) or contracted (blue) in corresponding *Colletotrichum* species. (**C**) Sankey diagram summarizing annotations of described secreted proteins. Columns, from left to right, denote orthologs accession, Pfam domain, effector prediction, and PHI-base annotation. (**D**) Counts of described secreted proteins in JDA-12 and their homologs in the other *Colletotrichum* species. The Red four-pointed star represent *C. siamense* JDA-12.

Fungal pathogens deploy a diverse secretome to overcome host defenses and promote infection. In this study, the number of predicted secreted proteins (SPs) across 18 *Colletotrichum* species ranged from 1135 to 1899, with species in Clade I (e.g., *C. fructicola* Nara_gc5 and JDA-12) exhibiting significantly higher SP counts than those in Clade II (e.g., *C. incanum* MAFF_238704) (Figure 5A). EffectorP 3.0 predicted that 19–20% of SPs were apoplastic effectors and 5–13% were cytoplasmic effectors (Figure 5B), indicating a greater proportion of cytoplasmic effectors compared to apoplastic effectors. Additionally, *Colletotrichum* species in Clade I possess a markedly higher number of effectors than those in Clade II, particularly apoplastic effectors. PHI-base provides curated data on genes experimentally shown to affect pathogen–host interaction outcomes [19]. PHI-base annotation revealed that apoplastic effectors in the studied *Colletotrichum* species displayed greater functional diversity than cytoplasmic effectors, which were limited to categories such as ‘unknown’, ‘effector’, ‘reduced virulence’, ‘increased virulence’, and ‘loss of pathogenicity’ (Figure 5C). Fungal effectors, typically characterized as being cysteine-rich, play a pivotal role in subverting plant immune responses [20]. Therefore, it was confirmed that the cysteine content of predicted effectors was significantly higher than that of other SPs and non-SPs (Figure 6A), as expected. Furthermore, apoplastic effectors in Clade I species typically exhibit much higher cysteine content than cytoplasmic effectors, while this trend is less evident in Clade II species.

Integrative analysis revealed that 15 secretome-related orthogroups, comprising 34 genes, were significantly expanded in JDA-12 (Figure 6B). In contrast, other species within Clade I, aside from *C. fructicola* Nara_gc5, exhibited limited expansions of secretome-related orthogroups, ranging from 1 to 13 genes, while Clade II displayed no significant contractions of orthogroups. In JDA-12, the significantly expanded secretome-related orthogroups included genes encoding AbiEii domain-containing proteins (OG0000020), putative cytoplasmic effectors devoid of Pfam domains (OG0000064), and enterotoxin_a domain-containing proteins (OG0000136) (Figure 6C,D, Table S3). Additional expansions included carbohydrate-active enzyme (CAZyme) domains such as Glyco_hydro_18, Glyco_hydro_cc, and Pectate_lyase_3, indicating potential associations with host cell wall degradation. Collectively, the expansion of these secreted protein families suggests that JDA-12 may possess an enhanced repertoire of secreted molecules involved in host interaction and extracellular adaptation, including potential roles in manipulating host cellular processes, modulating plant immune responses, or participating in extracellular interactions.

### 2.5. Expansion of the CBM24-GH71 CAZyme Subfamily in C. siamense JDA-12

**Figure 7:**
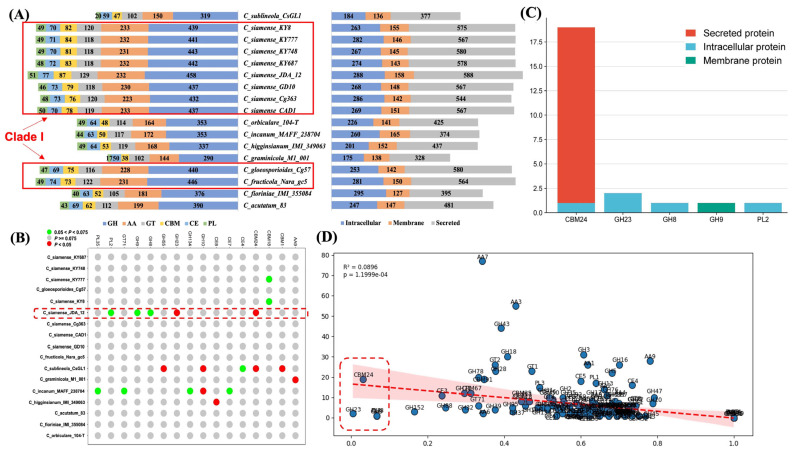
Comprehensive CAZyme analysis across *Colletotrichum* genomes presented in this study. (**A**) Distribution of CAZy enzyme classes per genome: GH, AA, GT, CBM, CE and PL. (**B**) Enrichment of CAZy families in each genome assessed via Fisher’s exact test. Red dots indicate statistically significant enrichment (*p*-value < 0.05) and green dots indicate moderate enrichment (0.05 ≤ *p*-value < 0.075). (**C**) Counts of proteins from CAZy subfamilies enriched in JDA-12 and their predicted subcellular localization. (**D**) Domain pattern of CBM24-containing proteins of JDA-12.

Carbohydrate-Active Enzymes (CAZymes) play crucial roles in fungal pathogenicity by degrading plant cell walls. A comparative analysis of CAZyme repertoires across 18 *Colletotrichum* genomes revealed marked variation, with gene counts ranging from 641 in *C. graminicola* M1.001 to 1034 in *C. siamense* JDA-12 (Figure 7A, Table S4), consistent with earlier studies [21]. Species within Clade I generally encoded more CAZymes than those in Clade II, suggesting an evolutionary expansion that may enhance their carbohydrate metabolic capabilities during interaction with the host plant. Notably, 47% to 59% of the CAZyme were also annotated as secreted proteins, highlighting their potential extracellular function and involvement in host tissue degradation.

Enrichment analysis using Fisher’s exact test identified CBM24 (*p*-value = 0.035) and GH23 (*p*-value = 1.45 × 10^−7^) as significantly overrepresented in JDA-12, with GH8, GH9, and PL2 showing moderate enrichment (0.05 ≤ *p*-values < 0.075) (Figure 7B, Table 3). Specifically, the CBM24 subfamily includes 19 members, of which 18 were annotated as secreted proteins (Figure 7C), indicating their likely secretion into the extracellular environment. Interestingly, structural domain analysis further revealed a conserved architecture featuring N-terminal CBM24 and C-terminal GH71 domains, indicative of a functionally integrated multi-domain structure (Figure 7D). Additionally, CBM1, CBM24, GH10, and GH55 were significantly enriched in *C. sublineola* CsGL1; GH10 in *C. incanum* MAFF 238704; AA9 in *C. graminicola* M1.001; and CE8 in *C. higginsianum* IMI 349063.

### 2.6. Investigation of Secondary Metabolite Biosynthesis Gene Clusters Unique to C. siamense JDA-12

**Figure 8:**
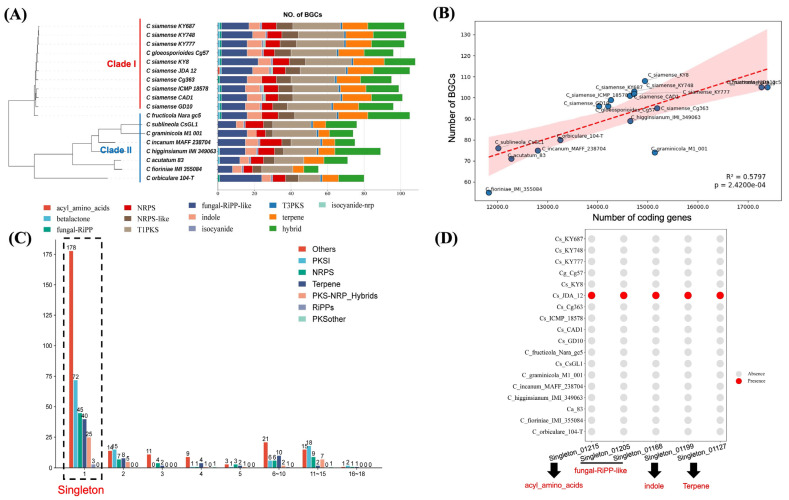
Secondary metabolite biosynthetic gene cluster (BGC) profiling across *Colletotrichum* genomes sequenced in this study. (**A**) Classification histogram showing the number of predicted BGCs per genome. (**B**) Scatterplot describing Pearson correlation between number of coding genes and BGCs. (**C**) Histogram illustrating the number of gene cluster families (GCFs) and singletons unique to individual genomes. (**D**) Presence/absence heatmap of five singleton BGCs uniquely identified in the JDA-12 genome.

Secondary metabolite (SM) biosynthetic genes in fungi are typically organized into biosynthetic gene clusters (BGCs), which consist of a core biosynthetic gene flanked by genes encoding transporters and tailoring enzymes [22,23]. In this study, we predicted 1632 BGCs across 18 *Colletotrichum* genomes using antiSMASH v5.1.1 in relaxed mode, with each genome harboring between 55 and 108 BGCs (Table S5). All analyzed Colletotrichum genomes exhibited abundant T1PKS, fungal RiPP-like, and terpene BGCs, averaging 22, 14, and 12 per genome, respectively (Figure 8A; Table S5). Notably, *C. siamense* strains KY8 and JDA-12 displayed the highest BGC counts (108 and 105, respectively), while *C. fioriniae* IMI 355084 had the fewest (55). Species belonging to Clade I consistently exhibited significantly higher BGC abundance compared to Clade II (Figure 8A). Moreover, the number of BGCs showed a positive correlation with the total gene count (R^2^ = 0.57, *p*-value = 0.000242) (Figure 8B), suggesting that the genome-size-driven expansion of secondary metabolism may enhance the ecological adaptability and elevated virulence of Clade I species within the CGSC.

The clustering of all predicted BGCs using BiG-SCAPE resulted in the identification of 196 gene cluster families (GCFs) and 363 singletons, with the latter representing species- or strain-specific BGCs. This classification included 74 ‘Other’ GCFs (comprising 475 BGCs), 31 non-ribosomal peptide synthetase (NRPS) GCFs (221 BGCs), 43 polyketide synthase type I (PKS-I) GCFs (326 BGCs), 29 terpene GCFs (174 BGCs), 2 other PKS GCFs (15 BGCs), 16 hybrids of PKS and NRPS (114 BGCs), and one ribosomally synthesized and post-translationally modified peptide (RiPP) GCF (8 BGCs) (Figure 8C). The predominance of singleton BGCs indicates a diverse secondary metabolite (SM) pathway among different *Colletotrichum* species. Further analysis identified five singleton BGCs unique to JDA-12 (Figure 8D; Figure S2): Singleton_01127, Singleton_01215, and Singleton_01199 are putatively involved in the biosynthesis of terpenes, acyl-amino acids, and indoles, respectively, while Singleton_01168 and Singleton_01205 were classified as fungal RiPP-like clusters (Table S6). These lineage-specific BGCs may represent novel or strain-specialized metabolic pathways, potentially contributing to ecological adaptation or host interaction.

Together, comparative genomic analyses identified several lineage-specific genomic features associated with extracellular interactions, carbohydrate utilization, and specialized metabolism in JDA-12. To determine whether JDA-12 exhibits enhanced pathogenic performance under strawberry-associated conditions, we further evaluated its pathogenic behavior across different strawberry cultivars and environmental scenarios.

### 2.7. Pathogenicity of Transformant C-4 on Strawberry Cultivars with Differential Resistance

**Figure 9:**
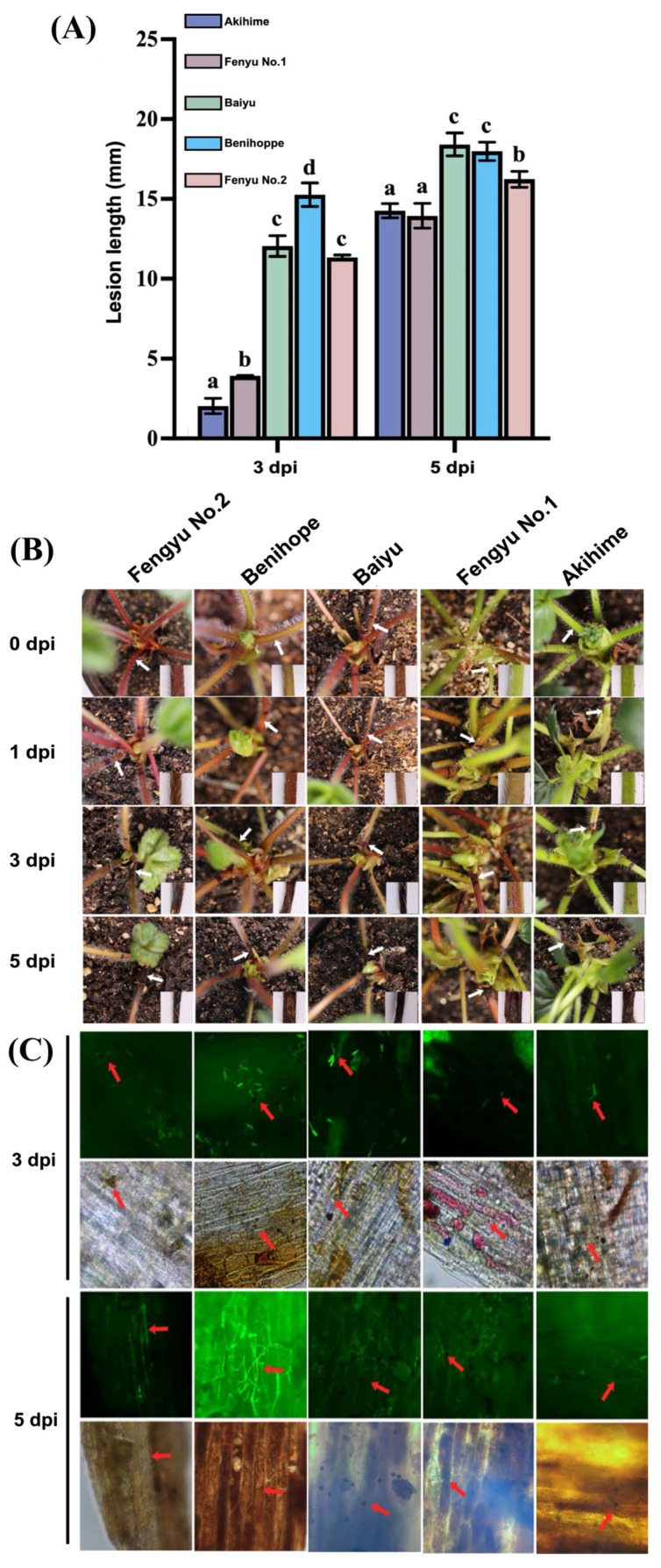
Pathogenicity of C-4 transformants on different strawberry cultivars under wounded and unwounded conditions. (**A**) Lesion length on five strawberry varieties (Akihime, Fenyu No. 1, Baiyu, Benihoppe, and Fenyu No. 2) at 3 and 5 days post-inoculation (dpi). Error bars represent the standard error, and different letters above bars indicate statistically significant differences (ANOVA test, *p*-value < 0.05). (**B**) Macroscopic disease symptoms on crowns of different strawberry cultivars at 0, 1, 3, and 5 dpi. White arrows indicate the inoculation sites or developing lesions. Insets show magnified views of the inoculation sites. (**C**) Microscopic observation of C-4 colonization and host response in strawberry crowns at 3 and 5 dpi. The upper image in each pair shows hyphae or conidia of C-4, while the lower image shows brightfield microscopy of the host tissue, highlighting fungal structures or host responses (red arrows).

To evaluate the pathogenicity of transformant C-4, five strawberry cultivars widely grown in Zhejiang Province (‘Akihime’, ‘Benihoppe’, ‘Baiyu’, ‘Fenyu No.1′, and ‘Fenyu No.2′) were inoculated under both non-wounded and wounded conditions. At 3 days post-inoculation (dpi), only mild lesions were observed on the crown of ‘Akihime’ and ‘Baiyu’ (0.72 ± 0.08 mm and 0.48 ± 0.15 mm, respectively) (Figure 9A). By 5 dpi, lesions developed in all cultivars, with ‘Benihoppe’ exhibiting the largest lesions (7.52 ± 0.74 mm), indicating the highest susceptibility, while ‘Fenyu No.1′ displayed minimal lesions (0.33 ± 0.04 mm), suggesting strong resistance. Under wounded conditions, rapid lesion development was detected at 3 dpi in ‘Benihoppe’, ‘Baiyu’, and ‘Fenyu No.2′ (12.05–15.26 mm), while ‘Akihime’ and ‘Fenyu No.1′ exhibited smaller lesions. Lesion progression continued to 5 dpi, with ‘Benihoppe’ and ‘Baiyu’ maintaining the largest necrotic areas (≥17.98 mm) (Figure 9B). Confocal microscopy further elucidated the infection dynamics (Figure 9C): under non-wounded conditions, conidial germination and appressoria formation were delayed in resistant cultivars (e.g., ‘Fenyu No.1′), while early infection structures were evident in susceptible cultivars. Under wounded conditions, advanced colonization, including branched hyphae and secondary conidia, was observed in ‘Benihoppe’ by 5 dpi, indicating accelerated infection progression. Collectively, these results underscore cultivar-dependent variation in susceptibility, with ‘Fenyu No.1′ exhibiting the highest resistance and ‘Benihoppe’ being the most susceptible to transformant C-4.

### 2.8. Effects of Environmental Factors on the Pathogenicity of Transformant C-4

**Figure 10:**
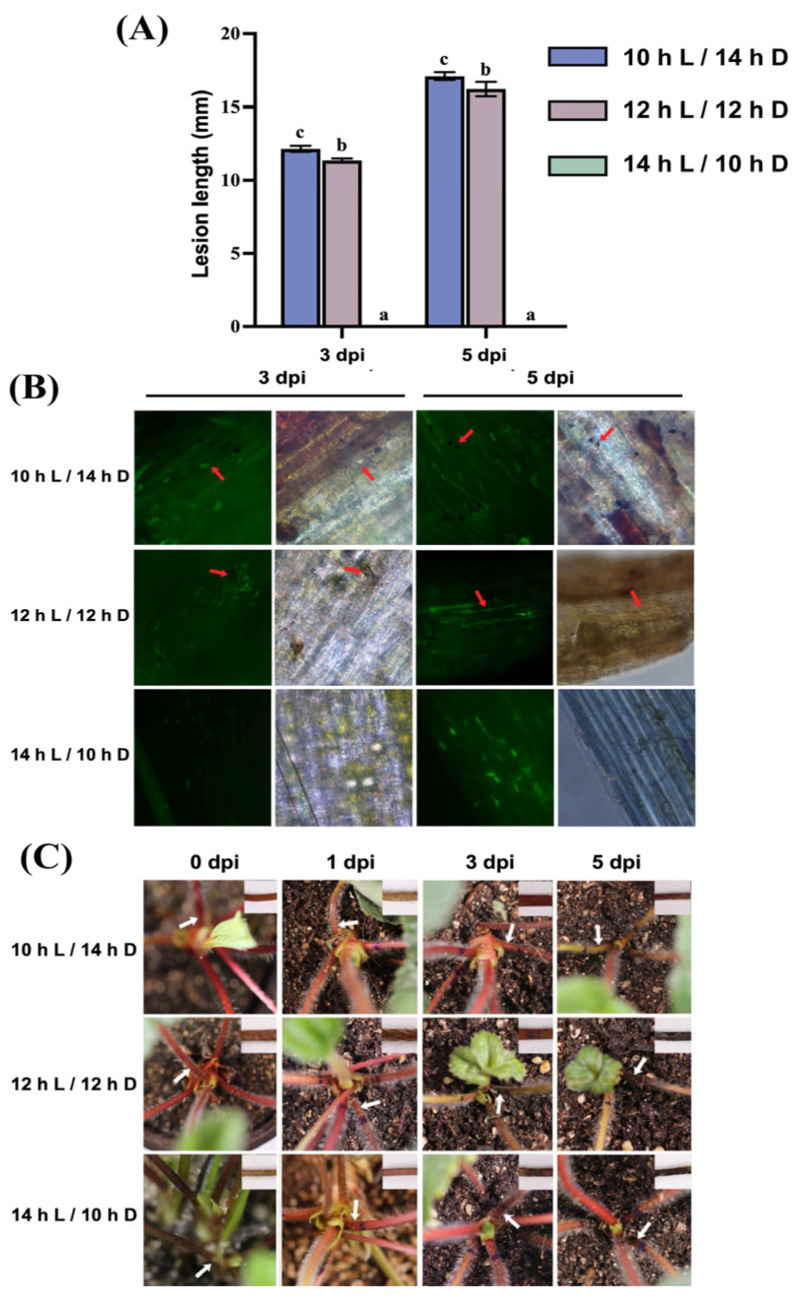
Effect of photoperiod on the pathogenicity of C-4 in strawberry. (**A**) Lesion length on ‘Fenyu No.2′at 3 and 5 dpi under conditions of (10L:14D), (12L:12D) and (14L:10D). Error bars represent the standard error, and different letters above bars indicate statistically significant differences (ANOVA test, *p*-value < 0.05). (**B**) Macroscopic SCR disease symptoms on crown of different strawberry cultivars at 0, 1, 3, and 5 dpi. Red arrows indicate the inoculation sites or developing lesions. Insets show magnified views of the inoculation sites. (**C**) Microscopic observation of C-4 colonization and host response in strawberry crowns at 3 and 5 dpi. The upper image in each pair shows hyphae or conidia of C-4, while the lower image shows brightfield microscopy of the host tissue, highlighting fungal structures or host responses (White arrows).

**Figure 11:**
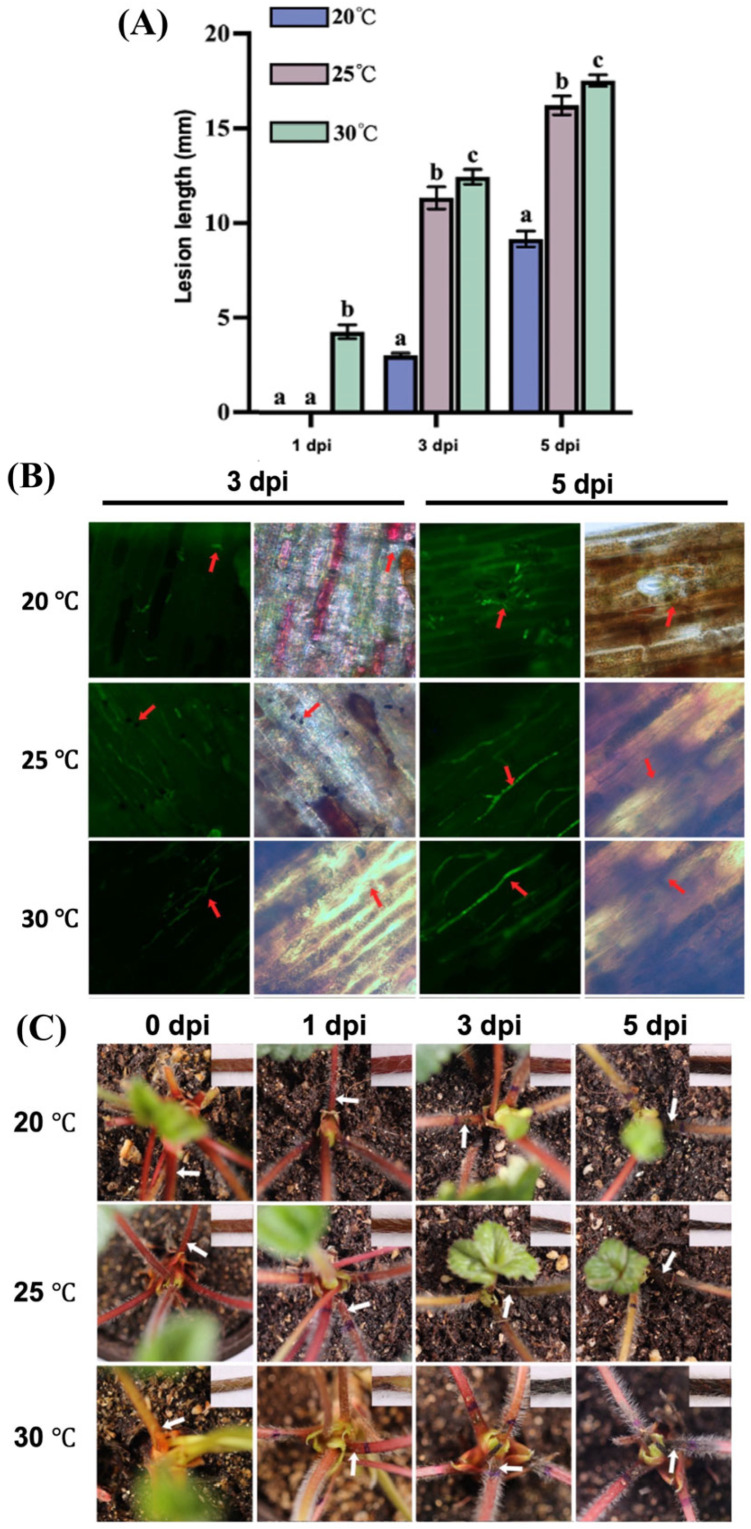
Effect of temperature on the pathogenicity of C-4 in strawberry. (**A**) Lesion length on ‘Fenyu No.2′at 1, 3 and 5 dpi under conditions of 20 °C, 25 °C and 30 °C. Error bars represent the standard error, and different letters above bars indicate statistically significant differences (ANOVA test, *p*-value < 0.05). (**B**) Macroscopic SCR disease symptoms on crown of different strawberry cultivars at 0, 1, 3, and 5 dpi. Red arrows indicate the inoculation sites or developing lesions. Insets show magnified views of the inoculation sites. (**C**) Microscopic observation of C-4 colonization and host response in strawberry crowns at 3 and 5 dpi. The upper image in each pair shows hyphae or conidia of C-4, while the lower image shows brightfield microscopy of the host tissue, highlighting fungal structures or host responses (White arrows).

**Figure 12:**
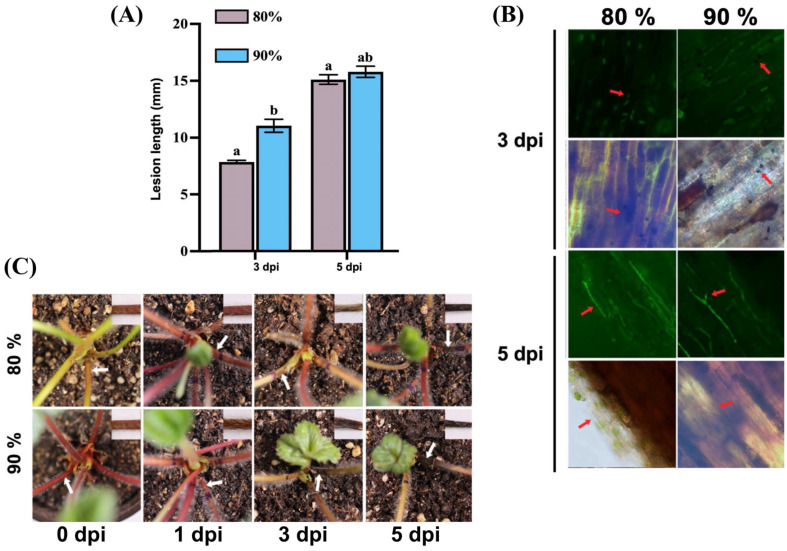
Effect of humidity on the pathogenicity of C-4 in strawberry. (**A**) Lesion length on ‘Fenyu No.2′at 1, 3 and 5 dpi under conditions of 80% and 90% relative humidity. Error bars represent the standard error, and different letters above bars indicate statistically significant differences (ANOVA test, *p*-value < 0.05). (**B**) Macroscopic SCR disease symptoms on crown of different strawberry cultivars at 0, 1, 3, and 5 dpi. Red arrows indicate the inoculation sites or developing lesions. Insets show magnified views of the inoculation sites. (**C**) Microscopic observation of C-4 colonization and host response in strawberry crowns at 3 and 5 dpi. The upper image in each pair shows hyphae or conidia of C-4, while the lower image shows brightfield microscopy of the host tissue, highlighting fungal structures or host responses (White arrows).

Environmental conditions profoundly influence the pathogenicity of the *C. siamense* transformant C-4 by modulating lesion development and the differentiation of infection structures. Under wounded conditions, lesion lengths at 3 and 5 days post-inoculation (dpi) reached 14.82 ± 0.61 mm and 17.06 ± 0.53 mm, respectively, under short-day (10L:14D) photoperiods, and 12.03 ± 0.44 mm and 15.34 ± 0.46 mm under intermediate (12L:12D) regimes. In contrast, long-day conditions (14L:10D) completely suppressed lesion formation (Figure 10A). Microscopic analysis confirmed that short photoperiods facilitated rapid development of germ tubes, appressoria, and primary hyphae within 3–5 dpi, whereas no visible infection structures formed under prolonged light exposure (Figure 10B). Temperature also significantly affected disease progression: lesion lengths at 30 °C reached 15.41 ± 0.38 mm (3 dpi) and 18.27 ± 0.62 mm (5 dpi), significantly exceeding those at 25 °C (10.14 ± 0.72 mm and 13.09 ± 0.57 mm) and 20 °C (3.96 ± 0.48 mm and 5.82 ± 0.71 mm) (Figure 11A). Correspondingly, high temperatures enhanced the formation of secondary hyphae and promoted tissue rot and necrosis (Figure 11B,C). Similarly, at 90% relative humidity, lesion lengths reached 13.72 ± 0.41 mm and 16.58 ± 0.35 mm at 3 and 5 dpi, respectively, compared to 7.86 ± 0.53 mm and 10.39 ± 0.67 mm at 80% humidity (Figure 12A). High humidity also accelerated appressoria development and secondary hyphal expansion, leading to more pronounced tissue browning and collapse (Figure 12B,C). Taken together, these results indicate that short photoperiods, elevated temperatures (~30 °C), and high relative humidity (≥90%) synergistically enhance the pathogenicity of C-4 by accelerating infection structures and host colonization, underscoring the pivotal role of environmental factors in promoting disease outbreaks under conducive climatic conditions.

## 3. Discussion

Strawberry (*Fragaria* × *ananassa*), increasingly cultivated for its softness and sweetness, is now severely threatened by crown rot, a globally destructive disease that causes seedling mortality, stunts plant growth, reduces yields, and leads to substantial economic losses [24,25,26]. Strawberry Crown Rot (SCR) represents a significant threat to the strawberry industry, with the primary causal agent identified as *C. siamense* [12], a pivotal member of the *C. gloeosporioides* species complex (CGSC) [18]. This pathogen has been reported across multiple strawberry-producing regions worldwide, and is recognized as the dominant pathogen responsible for crown rot in the Asia-Pacific region, including China, Korea, and Bangladesh [27,28,29]. This study initially collected SCR samples from diverse locations (Jian-de, Zhoushan and Lin’an) in Zhejiang Province, China, revealing a characteristic symptom ranging from incipient reddish crown lesions and associated young leaf wilting to advanced necrosis and ultimate plant demise, consistent with our previous research [14]. Subsequent microbial isolation identified *C. siamense* JDA-12 as the dominant pathogen with the strongest pathogenicity among all isolated pathogens, reflecting its host adaptation strategies within the corresponding agroecosystems and indicating a strong evolutionary fit to the cultivated strawberry in Zhejiang Province.

Phylogenetic analysis revealed that the species mentioned in the present study were divided into two main clades (Figure 2): Clade I, predominantly comprising taxa from the *C. gloeosporioides* Species Complex (CGSC), and Clade II, which harbored diverse species from distinct complexes, including *C. acutatum*, *C. graminicola*, *C. spaethianum*, and *C. orbiculare*. Notably, the topological structure of the species tree in this study exhibited congruence with a previous publication (ref. [30]). Interestingly, a robust whole-genome assembly for *C. siamense* strain JDA-12 revealed a genome size of 64.16 Mb (Table 1), which is markedly greater than that of previously documented *C. siamense* strains. This striking divergence suggests that strain JDA-12 has undergone substantial genome expansion. To explore the biological significance of this expansion, we examined gene families potentially associated with host colonization, ecological adaptation, and pathogenicity. To explore the potential relationship between the expansion of JDA-12 and its pathogenicity, we examined variations in the size of gene families potentially related to fungal virulence.

Notably, ortholog analysis revealed that 11 AbiEii-containing proteins were specifically expanded in JDA-12 and predicted to be secreted (Figure 4, Table S1). AbiEii has been characterized as a component of bacterial type IV toxin–antitoxin systems involved in abortive infection against bacteriophages [31,32]. However, the biological functions of AbiEii-containing proteins in fungi remain unclear. Similar cases of bacterial-derived or horizontally acquired genes contributing to lineage-specific innovations have been reported in fungal genomes, although the functional consequences of such gene acquisitions often require experimental validation. Therefore, the expansion of AbiEii-containing proteins in JDA-12 may represent a lineage-specific innovation rather than a direct functional equivalent of bacterial Abi systems. The expansion of this protein family in JDA-12 therefore suggests lineage-specific diversification rather than a directly demonstrated functional analogy with bacterial Abi systems. In this study, the predicted secretion of these expanded AbiEii-containing proteins indicates their potential localization in extracellular environments. Since secreted proteins can participate in diverse biological interactions, these proteins may represent candidate factors associated with host-associated adaptation or environmental responses. Nevertheless, whether AbiEii-containing proteins contribute to microbial interactions, host colonization, or other ecological processes in JDA-12 requires further experimental investigation. Their contribution to ecological fitness may involve interactions with diverse microorganisms or environmental factors in the strawberry-associated niche; however, direct evidence linking AbiEii expansion to competitive advantages or enhanced pathogenicity is currently unavailable.

In addition, other expanded gene families in JDA-12 may also contribute to its host adaptation. Among these, 37 expanded orthologs containing the Methyltransf_23 domain were identified as putative DNA methyltransferases. DNA methylation is known to regulate fungal development and pathogenicity [33]. In *Magnaporthe oryzae*, dynamic DNA methylation changes influence developmental processes, particularly asexual reproduction [34]. In *Botrytis cinerea*, BcDIM2 and BcRID2 synergistically regulate fungal development and virulence, with double mutants exhibiting impaired growth and reduced pathogenicity [35]. Similarly, reduced DNA methylation in *C. gloeosporioides* promotes the expression of pectin-degrading enzyme genes and enhances mango infection [36]. Therefore, the expansion of DNA methyltransferase-related genes in JDA-12 may represent an additional regulatory mechanism during adaptation to strawberry hosts.

Comparison of CAZyme profiles revealed a significant enrichment of the CBM24 subfamily in JDA-12 compared to its close relatives. Domain organization analysis indicated that these proteins exhibit a multi-domain architecture, featuring an N-terminal CBM24 domain and a C-terminal GH71 domain (Figure 7). According to dbCAN3 annotations, the CBM24 domain binds α-1,3-glucan but lacks catalytic activity. It is frequently associated with glycoside hydrolase domains that facilitate substrate targeting. In several fungi, including *Sclerotinia sclerotiorum*, *Botrytis cinerea*, and *Paradendryphiella salina*, GH71 exhibits α-1,3-glucanase activity. It is frequently coupled with the CBM24 domain to promote efficient degradation of α-1,3-glucans [37,38]. Interestingly, the cell wall of filamentous fungi comprises a core scaffold of branched β-1,3-glucan and chitin. This scaffold is surrounded by a matrix of amorphous polysaccharides, including α-1,3-glucan, whose composition varies among fungal lineages [39]. Surface α-1,3-glucan is considered a plant-refractory polysaccharide. It likely contributes to fungal evasion of host PAMP-triggered immunity by masking key structure cell wall components, such as chitin [40]. Additionally, α-1,3-glucan shields fungal cell walls from plant antifungal enzymes, thereby enhancing pathogen survival during infection, as experimentally verified in *Magnaporthe oryzae* [41]. Overall, we hypothesize that the expansion of CBM24-GH71 containing proteins in JDA-12 contributes to dynamic remodeling of its own cell wall. By potentially modifying α-1,3-glucan-associated cell wall structures, these proteins may influence the exposure of immunogenic components such as chitin and thereby affect host immune recognition. This mechanism may represent one possible strategy facilitating fungal persistence during host colonization, although direct evidence linking CBM24-GH71 expansion to strawberry immune suppression is still lacking.

A comparative analysis of SM biosynthesis clusters revealed that Colletotrichum species from Clade I, particularly those associated with CGSCs, possess a significantly higher number of BGCs compared to those from Clade II. This finding indicates a stronger secondary metabolite biosynthetic potential of *Colletotrichum* species from Clade I. The majority of species-specific BGC clustering highlighted a diverse landscape of secondary metabolites among *Colletotrichum* species in this study. Notably, JDA-12 contained five lineage-specific BGCs, including two fungal RiPP-like clusters, one terpene cluster, one acyl amino acid cluster, and one indole cluster. Previous studies have shown that *C. siamense* produces distinct terpenoids during host infection [42]. For instance, clavaric acid inhibits squalene synthase, thereby disrupting sterol biosynthesis in host cells. This process destabilizes plant cell membranes, and may facilitate fungal invasion [13]. Acyl-amino acid compounds have rarely been reported in plant pathogenic fungi. However, N-acylated aromatic amino acids, such as N-acyl dehydrotyrosine, have been identified in marine Planctomycetes. These compounds exhibit antimicrobial activity and may also function as signaling functions [43]. Based on these observations, the singleton acyl-amino acid (Singleton_01215) identified in JDA-12 may produce structurally related metabolites. Such metabolites might act as phytotoxins that facilitate the rapid cellular collapse and tissue necrosis observed during our pathogenicity assays on strawberry crowns.

Recent studies have demonstrated that fungal ribosomally synthesized and post-translationally modified peptides (RiPPs) represent an emerging class of specialized metabolites with diverse biological activities. For example, ustiloxin B, a cyclic RiPP produced by filamentous fungi, inhibits tubulin polymerization and disrupts microtubule assembly, resulting in strong cytotoxic effects [44]. Similarly, phomopsins are RiPP-derived antimitotic peptides that bind tubulin and interfere with microtubule formation [45]. These findings indicate that fungal RiPP metabolites can generate potent bioactive compounds that may contribute to plant-pathogen interactions by targeting host structural proteins to facilitate aggressive tissue colonization. Previous studies have identified RiPP-type BGCs in the Kahawae clade of CGSC [46]. In contrast, our study identified two lineage-specific RiPP-like BGCs in *C. siamense* JDA-12, a member of the Musae clade. Although the metabolites encoded by these two JDA-12-specific RiPP-like clusters remain unidentified, known fungal RiPP metabolites, such as ustiloxin B and phomopsins, demonstrate that this class of compounds can possess potent biological activities. Therefore, these clusters represent promising candidates for future functional characterization rather than confirmed virulence-associated pathways. This difference in RiPP repertoire between CGSC lineages may reflect species-specific diversification of RiPP-derived natural products within the genus *Colletotrichum*.

Beyond individual candidate virulence factors, successful colonization by *C. siamense* likely requires coordinated manipulation of host immunity through multiple molecular strategies. Collectively, the genomic features identified in JDA-12 suggest that this strain may employ multiple potential strategies to modulate host immune perception, including alteration of fungal surface properties, secretion of candidate interaction proteins, and production of specialized metabolites. Although direct experimental evidence is still lacking, these genomic characteristics provide possible explanations for how JDA-12 may enhance persistence and colonization within strawberry tissues. First, the expansion of secreted proteins, including cytoplasmic effector candidates and AbiEii domain-containing proteins, suggests an enhanced capacity to deliver molecules that may interfere with host cellular processes and immune signaling. Second, the enrichment of CBM24-GH71-containing proteins may contribute to fungal cell wall remodeling. Because α-1,3-glucan can mask immunogenic cell wall components such as chitin and reduce recognition by plant immune receptors, expansion of these proteins may influence host immune perception by regulating fungal surface properties during infection. Since α-1,3-glucan can mask immunogenic fungal molecules such as chitin and reduce recognition by plant immune receptors, expansion of these proteins may indirectly influence host immune perception during infection. Third, lineage-specific RiPP-like BGCs identified in JDA-12 may encode specialized metabolites with potential roles in host–microbe interactions. Although their products remain unidentified, fungal RiPP-derived compounds have been shown to possess cytotoxic and antimicrobial activities, suggesting possible roles in host–microbe interactions.

Previous research had systematically evaluated the virulence variability of *C. gloeosporioides* isolates on various strawberry cultivars grown in Florida [47,48]. However, the pathogenic profile of *C. siamense* across diverse strawberry cultivars remains poorly characterized. In this study, we investigate the virulence of JDA-12 on five major strawberry cultivars grown in Zhejiang Province. The findings demonstrate significant differences in cultivar susceptibility, with ‘Benihoppe’ being highly susceptible and ‘Fenyu No.1′ exhibiting comparatively lower disease severity and higher resistance to JDA-12 infection. The relatively higher resistance of ‘Fenyu No.1′ highlights its potential value as a resistant cultivar for inclusion in integrated management programs against strawberry crown rot. However, its resistance should be further validated under field conditions and against diverse *C. siamense* populations.

Environmental factors play a critical role in modulating pathogen virulence. Temperature fluctuations affect not only the survival thresholds of different pathogens but also regulate toxin biosynthesis and the expression of virulence-related genes [49]. High humidity facilitates spore formation in pathogens such as *Magnaporthe oryzae* [50]. It also promotes pathogen adhesion and penetration while suppressing salicylic acid- and ethylene-mediated host immunity. Additionally, light regulates the rhythms of conidiation and spore release in *Neurospora crassa* and *Podosphaera pannosa* [51,52]. Light also influences secondary metabolism and the balance between sexual and asexual development in *Aspergillus nidulans* [53]. Our results showed that shorter photoperiods (10L:14D and 12L:12D), high temperature (approximately 30 °C), and high humidity (90%) significantly promoted the development of infection structures. These environmental conditions also accelerated disease progression. Together, these environmental conditions enhanced disease development by promoting infection structure formation and host colonization, suggesting that environmental compatibility is an important component of JDA-12 adaptation to strawberry-associated environments.

This study systematically isolated pathogens from strawberry crown rot samples collected in Zhejiang, China, and confirmed that *C. siamense* strain JDA-12 represents a predominant and highly adapted pathogen associated with SCR disease in this region. Comparative genomic analyses with related *Colletotrichum* species identified lineage-specific expansions of AbiEii-containing proteins, CBM24-GH71 CAZyme proteins, and RiPP-like biosynthetic gene clusters, which may represent genomic signatures associated with ecological fitness and host-associated adaptation of JDA-12. Rather than representing individual virulence determinants, these lineage-specific genomic features provide a genomic framework for understanding the ecological fitness and host-associated adaptation of JDA-12. The pathogenicity assays further revealed that JDA-12 exhibits strong host colonization capability under specific strawberry-associated conditions, providing phenotypic evidence consistent with its genomic features associated with ecological adaptation. In addition, environmental assays demonstrated that short photoperiods, elevated temperatures, and high humidity promote disease development by facilitating infection structure formation and host colonization. Collectively, these findings improve our understanding of the genomic basis underlying the ecological success and pathogenic potential of *C. siamense* JDA-12 and provide useful information for SCR disease management.

## 4. Materials and Methods

### 4.1. Fungal Materials, Plant Materials and Culture Conditions

The wild-type strain JDA-12 of *C. siamense*, utilized in this study, was isolated from strawberry crown rot tissues collected in Jian-de, Zhoushan, and Lin’an, Zhejiang Province, China. The JDA-12 strain and its transformants were cultured on potato dextrose agar (PDA) medium and maintained at 4 °C for storage. PDA medium was prepared using 200 g potato, 20 g glucose, and 20 g agar per liter of distilled water. Healthy wild-type strawberry seedlings, six-week-old, including the cultivars ‘Benihoppe’, ‘Akihime’, ‘Baiyu’, ‘Fenyu No.1′, and ‘Fenyu No.2′, were kindly provided by the Hangzhou Academy of Agricultural Sciences.

### 4.2. DNA Extraction, Genome Sequencing and Assembly

JDA-12 was cultured on potato dextrose agar (PDA) plates at 25 °C for 10 days until the mycelium covered approximately two-thirds of the medium surface. Subsequently, the fungal cultures were harvested and immediately flash-frozen in liquid nitrogen, followed by thorough grinding with a pre-chilled mortar and pestle to obtain a fine mycelial powder. The resulting mycelial powder was transferred to a 2 mL microcentrifuge tube for genomic DNA extraction, which was performed using the Genomic DNA Extraction Kit (Sangon Biotech, Shanghai, China) according to the manufacturer’s recommended protocol. The purity and concentration of the extracted genomic DNA were assessed using a NanoDrop One spectrophotometer (Thermo Fisher Scientific, Wilmington, DE, USA). Whole-genome sequencing was subsequently conducted using the Oxford Nanopore Technologies (ONT) platform. To complement the genomic data and provide transcriptomic evidence, RNA sequencing was also performed on the Illumina HiSeq 4000 platform, utilizing total RNA extracted from mycelial samples cultured on PDA medium. The de novo genome assemblies were generated using NextDenovo v2.4.0 and NextPolish v1.3.1. Repeats were masked using RepeatMasker v4.1.2 with repeat library generated by RepeatModeler v2.01.

### 4.3. Collection and Annotation of Genome Assemblies

We retrieved 17 publicly available *Colletotrichum* genomes from the NCBI database, as follows: *C. acutatum* strain 83 (GCA_021124905.1), *C. fioriniae* IMI 355084 (GCF_027942845.1), *C. fructicola* strain Nara_gc5 (GCF_000319635.2), *C. gloeosporioides* strain Cg57 (GCA_040955525.1), *C. graminicola* strain M1.001 (GCF_000149035.1), *C. higginsianum* strain IMI 349063 (GCF_001672515.1), *C. incanum* strain MAFF 238704 (GCA_001625285.1), *C. orbiculare* strain 104-T (GCA_000350065.2), *C. siamense* strain CAD1 (GCA_013201865.1), *C. siamense* strain Cg363 (GCF_013390195.1), *C. siamense* strain GD10 (GCA_027406245.1), *C. siamense* strain ICMP 18578 (GCA_008520285.1), *C. siamense* strain KY687 (GCA_014705105.1), *C. siamense* strain KY748 (GCA_014705055.1), *C. siamense* strain KY777 (GCA_014705195.1), *C. siamense* strain KY8 (GCA_014705345.1), and *C. sublineola* strain CsGL1 (GCA_020631755.1). Together with *C. siamense* strain JDA-12 from this study, a total of 18 Colletotrichum species were analyzed. Gene annotation was performed on the repeat-masked assemblies using BRAKER2, which integrates evidence from relevant RNA-Seq data and homologous fungal proteins. The completeness of the genome assembly was evaluated using BUSCO v5.12 (fungi_odb9) based on the predicted gene models.

### 4.4. Ortholog Identification and Phylogenetic Analysis

Ortholog clustering was conducted using OrthoFinder v2.5.5, applying an *E*-value threshold of 1 × 10^−5^ and an MCL inflation index of 2.0. Multiple sequence alignments of single-copy orthologs were generated with MAFFT v7.490. Subsequently, a species phylogenetic tree was constructed using IQ-TREE v2.0.7 under default settings with automatic model selection based on the best-fitting substitution model. CAFÉ (Computational Analysis of Gene Family Evolution, v5.0) was utilized to compare the sizes of ortholog families across species to their respective most recent common ancestor (MRCA) using fossil data for species divergence times sourced from the TimeTree website (https://timetree.org, accessed on 30 April 2026). Rapidly evolving ortholog families were estimated with the best-fit global birth/death parameter (λ) set at 0.0001 and a *p*-value threshold of 0.01. The ultra-metric tree for each ortholog family was generated using r8s v1.81.

### 4.5. Pathogenicity Assay of C. siamense JDA-12 on Different Strawberry Cultivars

Four-week-old healthy strawberry seedlings from five cultivars (‘Akihime’, ‘Benihoppe’, ‘Baiyu’, ‘Fenyu No.1′, and ‘Fenyu No.2′) were selected for pathogenicity assessment. The concentration of the conidial suspension of the GFP-labeled transformant C-4 was adjusted to 1 × 10^6^ spores/mL using 2% (*w*/*v*) gelatin solution. Subsequently, 5 mL of the conidial suspensions was sprayed approximately 5 mm above the stolon, which were maintained in chambers (12 h light/12 h dark cycle) at 90% humidity and 25 °C. The assay was repeated three times, with five seedlings were inoculated from each cultivar for each repeat. Epidermal tissues were excised from the base of the strawberry crown using a scalpel at 1, 2, 3, 4 and 5 days post-inoculation (dpi). These tissues were placed on glass slides with 10% physiological glycerol (with 0.9 M NaCl). The infection process of C-4 on the strawberry epidermis was observed using a Nikon fluorescence microscope (Nikon Corporation, Tokyo, Japan). At 15 dpi, internal colonization by C-4 was further examined using an inverted laser scanning confocal microscope.

### 4.6. Assessment of the Effects of Photoperiod, Temperature, and Humidity on the Pathogenicity of C-4

Controlled inoculation experiments were conducted based on previously described methods. Photoperiod treatments were set at 10 h light/14 h darkness, 12 h light/12 h darkness, and 14 h light/10 h darkness under constant temperature of 25 °C and humidity of 90%. Temperature treatments were set at 20 °C, 25 °C, and 30 °C, with a fixed photoperiod of 12 h light/12 h darkness and 90% humidity. Humidity treatments were established at 80% and 90% under 12 h light/12 h darkness photoperiod and a constant temperature of 25 °C. For each treatment, six strawberry seedlings were inoculated, and three independent replicates were performed. Lesion lengths were observed and measured at 1, 3 and 5 days post-inoculation (dpi). Epidermal tissues from the base of the strawberry crown were prepared for observation using a Nikon fluorescence microscope.

## Figures and Tables

**Table 1:** Genomic Assembly Features of *C. siamense* JDA12 and Other Selected *Colletotrichum* Species.

Genome Assembly/Strain	Host	Assembly Size (Mb)	Contig Count	L50	N50 (bp)	Max Contig Length (bp)	Min Contig Length (bp)	GC Content	BUSCO Coverage	Number of Genes
*C. fioriniae* IMI_355084	*Quercus ilex*	49.45	46	6	2,983,733	7,342,820	485	36.33	95	11,830
*C. fructicola* Nara_gc5	*Fragaria x ananassa*	59.6	13	5	5,461,344	9,276,597	55,177	51.38	98.7	17,388
*C. gloeosporioides* Cg57	*Fragaria x ananassa*	57.77	2193	98	177,688	572,964	200	51	99	14,213
*C. graminicola* M1_001	*Zea mays*	57.49	14	5	6,444,216	7,652,868	67,326	41.64	97.1	15,139
*C. higginsianum* IMI_349063	*Brassica rapa* subsp. *chinensis*	50.72	25	5	5,201,688	6,042,495	17,011	52.51	98.4	14,651
*C. incanum* MAFF_238704	*Raphanus sativus* L. Daikon Group	53.6	2896	91	177,164	1,081,293	200	46.9	92.8	12,809
*C. orbiculare* 104-T	*Cucumis sativus*	89.75	355	13	2,078,754	5,815,657	200	20.91	96.7	13,253
*C. acutatum* 83	*Fragaria x ananassa*	49.75	1998	98	146,146	909,718	200	46.02	98.3	12,276
*C. sublineola* CsGL1	*Sorghum bicolor*	67.67	74	8	2,691,276	6,303,522	14,431	47.71	99.3	12,020
*C. siamense* CAD1	*Manihot esculenta*	58.4	308	22	881,809	2,798,639	204	40.52	99.2	14,645
*C. siamense* Cg363	*Fragaria x ananassa*	62.94	22	5	5,441,060	7,966,678	42,537	48.95	97.7	15,190
*C. siamense* GD10	*Mangifera indica*	56.19	14	5	4,998,354	7,602,691	335,449	51.43	99.1	14,027
*C. siamense* ICMP_18578	*Coffea arabica*	55.96	235	20	927,217	2,573,469	204	37.61	99.2	14,264
*C. siamense* KY8	*Malus domestica*	57.57	1595	215	77,820	358,189	201	53.16	98.1	14,945
*C. siamense* KY687	*Fragaria x ananassa*	57.44	697	91	207,360	681,595	203	51.83	98.8	14,722
*C. siamense* KY748	*Fragaria x ananassa*	57.08	1272	155	114,013	575,598	202	50.83	98.6	14,734
*C. siamense* KY777	*Prunus persica*	57.71	971	102	179,466	759,256	201	49.84	98.9	14,732
*C. siamense* JDA-12	*Fragaria x ananassa*	64.16	285	6	4,500,852	7,929,992	3428	50.68	98.3	17,268

**Table 2:** Classification of Orthologs generated by CAFE v5.

Species	Single-Copy Core Orthogroups	Accessory Orthogroups	Multi-Copy Core Orthogroups	Species-Specific Orthogroups	Unassigned Genes
*C. siamense* KY687	5788	7781	1097	31	25
*C. siamense* KY748	5788	7747	1116	33	50
*C. siamense* KY777	5788	7788	1098	34	24
*C. gloeosporioides* Cg57	5788	7284	1074	34	33
*C. siamense* KY8	5788	7896	1137	32	92
*C. siamense* JDA-12	5788	9263	1262	392	563
*C. siamense* Cg363	5788	8156	1097	7	142
*C. siamense* ICMP_18578	5788	7346	1091	2	37
*C. siamense* CAD1	5788	7643	1110	35	69
*C.siamense* GD10	5788	7069	1084	51	35
*C. fructicola* Nara_gc5	5788	9611	1149	355	485
*C. sublineola* CsGL1	5788	4399	1276	238	319
*C. graminicola* M1_001	5788	4405	1180	50	3716
*C. incanum* MAFF_238704	5788	4864	1311	260	586
*C. higginsianum* IMI_349063	5788	6082	1260	108	1413
*C. acutatum* 83	5788	5156	1156	40	136
*C. fioriniae* IMI_355084	5788	4715	1146	0	181
*C. orbiculare* 104-T	5788	4850	1167	91	1357

**Table 3:** CAZymes Subfamilies enriched in *C. siamense* JDA-12.

Family	*p*-Value	Gene Count in *C. siamense* JDA-12	Ratio of *p*_Value/Count	Gene Count in Other Species	Substrate
CBM24	0.035461465	19	535.7929	159	Alpha-glucan
GH23	1.45 × 10^−7^	2	13,824,204.7772	0	peptidoglycan
GH8	0.000200248	1	4993.8073	0	beta-glucan
GH9	0.000200248	1	4993.8073	0	cellulose, beta-glucan, beta-glucan
PL2	0.000200248	1	4993.8073	0	pectin

## Data Availability

The data presented in the study were deposited in the National Center for Biotechnology Information (NCBI) database with accession number PRJNA1281380.

## Supplementary Materials

The following supporting information can be downloaded at: https://www.mdpi.com/article/10.3390/plants15152366/s1.
